# Regulation of Skeletal Muscle Atrophy in Cachexia by MicroRNAs and Long Non-coding RNAs

**DOI:** 10.3389/fcell.2020.577010

**Published:** 2020-09-15

**Authors:** Rui Chen, Si Lei, Ting Jiang, Yanling She, Huacai Shi

**Affiliations:** ^1^Guangdong Traditional Medical and Sports Injury Rehabilitation Research Institute, Guangdong Second Provincial General Hospital, Guangzhou, China; ^2^Department of Radiology, The Third Affiliated Hospital, Sun Yat-sen University, Guangzhou, China

**Keywords:** skeletal muscle, atrophy, cachexia, miRNAs, lncRNAs

## Abstract

Skeletal muscle atrophy is a common complication of cachexia, characterized by progressive bodyweight loss and decreased muscle strength, and it significantly increases the risks of morbidity and mortality in the population with atrophy. Numerous complications associated with decreased muscle function can activate catabolism, reduce anabolism, and impair muscle regeneration, leading to muscle wasting. microRNAs (miRNAs) and long non-coding RNAs (lncRNAs), types of non-coding RNAs, are important for regulation of skeletal muscle development. Few studies have specifically identified the roles of miRNAs and lncRNAs in cellular or animal models of muscular atrophy during cachexia, and the pathogenesis of skeletal muscle wasting in cachexia is not entirely understood. To develop potential approaches to improve skeletal muscle mass, strength, and function, a more comprehensive understanding of the known key pathophysiological processes leading to muscular atrophy is needed. In this review, we summarize the known miRNAs, lncRNAs, and corresponding signaling pathways involved in regulating skeletal muscle atrophy in cachexia and other diseases. A comprehensive understanding of the functions and mechanisms of miRNAs and lncRNAs during skeletal muscle wasting in cachexia and other diseases will, therefore, promote therapeutic treatments for muscle atrophy.

## Introduction

Cachexia is a complex metabolic condition accompanied by progressive bodyweight loss, skeletal muscle atrophy, and decreased muscle strength. Muscle atrophy is an important factor associated with physical disability, poor quality of life, and increased mortality (Prado et al., 2008; Fearon et al., 2011). Cachexia is common in patients with certain cancers and other diseases in the advanced stages, such as patients with intensive care unit (ICU)-acquired (Garros et al., 2017) weakness or chronic obstructive pulmonary disease (COPD) (Man et al., 2009), chronic kidney disease (CKD) (Zhang et al., 2019), and heart failure (Anker et al., 1997). Patients with cachexia are less tolerant or responsive to interventions, which limits the treatment options and their efficacy, for example, the dose-limiting toxicity of radiation and chemotherapy in patients with cancer cachexia (de Castro et al., 2019).

Skeletal muscle atrophy in cachexia is due to a disruption in the balance between protein synthesis and degradation, which can be partially, but not fully reversed by nutritional support (Baracos et al., 2018; de Castro et al., 2019). Reduced protein levels result from decreased protein synthesis, increased degradation, and abnormalities in apoptosis or autophagy (Lok, 2015). Protein degradation pathways mainly include ubiquitin-proteasome pathway (UPP) and autophagy-lysosomal pathway (ALP). The ubiquitin-proteasome pathway is an active protein degradation pathway that degrades ubiquitinated proteins in cells. This way is mainly composed of ubiquitin, ubiquitin activating enzyme, ubiquitin conjugating enzyme, ubiquitin ligase (ubiquitinprotein ligase, E3), protease and its substrate. E3 specifically regulates target protein degradation by identifying and binding specific target protein sequences. In skeletal muscle, there are two muscle-specific E3 ligases: muscular atrophy fbox-1 protein (MAFbx/Atrogin-1) and muscle-specific RING finger protein -1 (MuRF-1). Atrogin-1 and MuRF-1 are key proteins leading to muscle protein degradation (Rom and Reznick, 2016). Models of muscle atrophy demonstrate a consistent increase in the expression level of Atrogin-1 and MuRF-1. Autophagy is a highly conserved process in eukaryotes that occurs in the cytoplasm, where excess or abnormal organelles are transported to lysosomes for degradation. There are three main types of autophagy in the cell, namely molecular chaperone-mediated autophagy, microautophagy and macrophage. And in our previous study, CoCl_2_-mimicked hypoxia induces autophagy in skeletal C2C12 myotubes, and increases the expression of muscle-specific ubiquitin ligase Atrogin-1, which reveals an important link between autophagy and muscle atrophy (Chen et al., 2017).

Whereas proteins are thought to be the dominant regulators of cell functions, a variety of non-protein-coding processes also operate. It is increasingly clear that the non-protein-coding parts of the genome are important for development, physiology, and disease progression (Sonkoly et al., 2005; Faghihi et al., 2008). microRNAs (miRNAs) and long non-coding RNAs (lncRNAs) are vital non-coding RNAs (ncRNAs). Studies have shown that miRNAs and lncRNAs may be associated with cancer (Ramalho-Carvalho et al., 2017), as well as neurological (Faghihi et al., 2008), cardiovascular (Ponnusamy et al., 2019), and other diseases (Iannone et al., 2020).

miRNAs and lncRNAs are also reported to play critical roles in muscle development and other muscle-related diseases, such as skeletal muscle wasting in cachexia (Chen et al., 2006, 2010; Li et al., 2017). miR-1 (Chen et al., 2006), miR-133 (Chen et al., 2006), miR-206 (Chen et al., 2010), and miR-29b (Li et al., 2017) can modulate muscle development and wasting. Our previous studies showed that 2,922 lncRNAs are differentially regulated during C2C12 differentiation, and these lncRNAs may be involved in multiple mechanisms regulating gene expression (Chen et al., 2018). In recent reports, linc-MD1 was shown to play a vital role through competing endogenous RNA (ceRNA) (Cesana et al., 2011), whereas lincRNA Yam-1 acted in *cis* (Lu et al., 2013), and lncRNA MUNC functioned in *trans* during skeletal muscle development (Cichewicz et al., 2018). A systematic review of our groups provided a summary of the known functions and mechanisms of lncRNAs in skeletal muscle development (Chen et al., 2020). However, 4,409 lncRNAs are differentially regulated by CoCl_2_ (Chen et al., 2019), which triggers atrophy in skeletal C2C12 myotubes (Chen et al., 2017), indicating the potential involvement of lncRNAs in muscle wasting.

To develop approaches to improve skeletal muscle mass, strength, and function, a more comprehensive understanding of the key pathophysiological processes leading to muscular atrophy is needed. Here, we summarize the known miRNAs and lncRNAs regulating skeletal muscle wasting in cachexia and other diseases. A comprehensive understanding of the functions and mechanisms of miRNAs and lncRNAs during muscle atrophy in cachexia will help identify potential therapeutic approaches for muscle atrophy.

## miRNAs Involved in Skeletal Muscle Atrophy

Although the mechanism of muscular atrophy has been investigated extensively, the key processes driving muscular atrophy in different diseases requires further clarification. miRNAs are short ncRNA molecules (18–25 nucleotides) that are vital negative regulators of gene expression. They fine-tune gene expression via post-transcriptional mechanisms, leading to inhibition of translation or degradation of target mRNAs, and are involved in many biological pathways. miRNAs play important roles in maintaining skeletal muscle homeostasis by regulating muscle metabolism, growth, and regeneration. Muscle-specific miRNAs have emerged as significant regulators of muscle wasting.

### miRNAs That Promote Skeletal Muscle Atrophy

**FIGURE 1:**
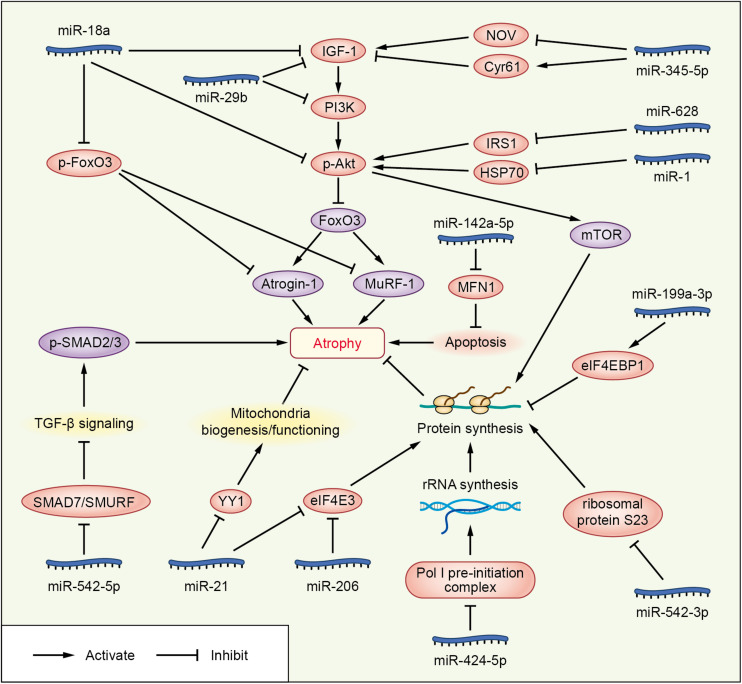
Functional miRNAs involved in promoting skeletal muscle wasting in cachexia. miRNAs are represented in blue; molecules directly targeted by the miRNAs are shown in pink; and other signaling molecules involved in muscle wasting are shown in purple.

Emerging studies have demonstrated that certain miRNAs promote muscle atrophy via the PI3K/Akt/mTOR signaling pathway (Figure 1 and Tables 1, 2). miR-29b promotes atrophy by targeting insulin-like growth factor 1 (IGF-1) and PI3K in response to different atrophic stimuli in cells and mouse models. Inhibition of miR-29b attenuates atrophy induced by dexamethasone (Dex), tumor necrosis factor (TNF)-α, and H_2_O_2_ treatment (Li et al., 2017). It was reported that miR-345-5p downregulated nephroblastoma overexpressed (NOV) and upregulated cysteine-rich 61 (Cyr61) in human skeletal muscle with cancer cachexia. NOV and Cyr61 are involved in the IGF-1, Akt, and mTOR pathways which promote protein synthesis, thus miR-345-5p may lead to muscle atrophy by suppressing protein synthesis (Narasimhan et al., 2017). In addition, miR-18a blocks PI3K/Akt signaling by direct inhibition of IGF-1 expression in a 3’UTR-dependent manner in C2C12 myotubes and in mice, and it also decreases the phosphorylation of Akt and FoxO3, thus increases the expression of Atrogin-1, MuRF-1, and cathepsin L, and eventually leads to myotube atrophy (Liu et al., 2017).

**TABLE 1:** Functional miRNAs involved in promoting skeletal muscle wasting in cell or rodent cachexia.

miRNAs	Partner	Model	References
miR-29b ↑	Insulin-like growth factor 1 (IGF-1) ↓, PI3K ↓	Mice (denervation, dexamethasone (Dex), fasting, aging, immobilization, cachexia in colon carcinoma); myoblasts (TNF-α and H_2_O_2_)	Li et al., 2017
miR-18a ↑	IGF-1↓	Mice and C2C12 myotubes of overexpression miR-18a	Liu et al., 2017
miR-1 ↑	HSP70 ↓	Dex-induced mice and C2C12 myotubes	Kukreti et al., 2013
miR-628 ↑	Insulin receptor substrate 1 (IRS1) ↓	Burn-injured rats	Yu et al., 2016
miR-206 and miR-21 ↑	YY1 ↓ and eIF4E3 ↓	Mice (starvation, denervation, diabetes, and cachexia in colon carcinoma)	Soares et al., 2014
miR-142a-5p ↑	Mitofusin-1 (MFN1) ↓	Denervation-induced mice	Yang et al., 2020

**TABLE 2:** Functional miRNAs involved in skeletal muscle wasting in human cachexia.

miRNAs	Partner	Model	Sample Numbers (Control/Cachectic)	Age (mean, in years) (Control/Cachectic)	References
miR-345-5p ↑	Nephroblastoma Overexpressed gene (NOV) ↓, cysteine-rich 61 (Cyr61) ↑	pancreatic or colorectal cancer	20/22	63.6 ± 7.9/64.9 ± 10.1	Narasimhan et al., 2017
miR-199a-3p ↑	eIF4EBP1↑	pancreatic or colorectal cancer	20/22	63.6 ± 7.9/64.9 ± 10.1	Narasimhan et al., 2017
miR-542-5p ↑	SMADs ↓	COPD, ICU-acquired weakness, and aortic surgery	COPD:12/24; ICU:7/17; Aortic surgery:19/21	67 ± 8/66 ± 10; 68 ± 11/62 ± 18; 58.7 ± 15.4/68.1 ± 14.9	Garros et al., 2017
miR-542-3p ↑	Ribosomal protein S23 ↓	COPD	16/52	66 ± 8/66 ± 8	Farre-Garros et al., 2019
miR-424-5p ↑	Pre-initiation complex (PIC) ↓	COPD, ICU-acquired wasting, aortic surgery and individuals from the Hertfordshire Sarcopenia Study	COPD:16/49 ICU: 7/17 aortic surgery: 19/21 Hertfordshire Sarcopenia Study:59/5	65 ± 8/66 ± 8; 68 ± 11/63 ± 17; 58.7 ± 15.4/68.1 ± 14.9 Not applicable	Connolly et al., 2018
miR-422a ↓	SMAD4 ↑ and TGF-β↑	COPD, ICU-acquired weakness undergoing aortic surgery	COPD:16/52 aortic surgery:19/21	65 ± 8/66 ± 8; 58.7 ± 15.4/68.1 ± 14.9	Paul et al., 2018

In addition to the PI3K/Akt/mTOR pathway, two miRNAs can enhance muscle wasting via Akt/FoxO signaling (Figure 1 and Table 1). Increasing miR-1 expression during Dex-induced atrophy reduces the level of HSP70, which can result in decreased phosphorylation of Akt, enhanced activation of FoxO3, upregulation of Atrogin-1 and MuRF-1, and progression of skeletal muscle atrophy (Kukreti et al., 2013). miR-628 promotes burn-induced skeletal muscle atrophy via targeting insulin receptor substrate 1 (IRS1). Rats subjected to burn injury exhibited skeletal muscle atrophy, significantly decreased IRS1 protein expression, and significantly increased skeletal muscle cell apoptosis. miR-628, a potential regulator of IRS1 protein translation, was also clearly elevated after the burn injury. In experiments in L6 myocytes, apoptosis was accelerated after induction of miR-628 expression, and the protein levels of IRS1 and p-Akt, involved in the IRS1/Akt/FoxO pathway, were decreased significantly. The levels of the apoptosis-related proteins FoxO3 and cleaved caspase 3 were also increased after induction of miR-628 expression (Yu et al., 2016).

Besides the above signaling pathways, some miRNAs facilitate cachexia muscle atrophy by regulating eukaryotic initiation factor 4E (eIF4E), one of the most critical factors regulating ribosome assembly and protein synthesis (Figure 1 and Tables 1, 2). miR-206 enhances wasting by decreasing the action of eIF4E3, and miR-21 inhibits the expression of transcription factor YY1 and eIF4E3, thus promoting muscle wasting in four different models: starvation, denervation, diabetes, and cancer cachexia induced by colon carcinoma (Soares et al., 2014). YY1 is reported to play an important role in the maintenance of mitochondrial functions, and its inactivation may contribute to exercise intolerance and mitochondrial myopathies (Blattler et al., 2012), promoting skeletal muscle wasting. miR-199a-3p affects the eIF4EBP1 gene and reduces the activity of the mTOR pathway, thus interfering with protein synthesis to promote muscle wasting in human skeletal muscle with cancer cachexia (Narasimhan et al., 2017). What’s more, in a meta-analysis, miR-199a is identified as a potential miRNA that inhibits Junb and Caveolin 1 expression, thus suppressing muscle wasting in cancer cachexia (Freire et al., 2019).

Additionally, miRNAs may contribute to muscle wasting via other pathways (Figure 1 and Tables 1, 2). Elevated miR-542-5p may cause muscle atrophy in ICU patients by activating SMAD2/3 phosphorylation. miR-542-5p increases the p-SMAD2/3 level by suppressing the inhibitory components of the TGF-β signaling pathway via a reduction in the expression of SMAD7 and SMURF1, inhibitors of TGF-β type I receptors, and by reducing the phosphatases (such as PPP2CA) that limit TGF-β signaling by dephosphorylating and inactivating SMADs (Garros et al., 2017). In other research, miR-542-3p suppressed ribosomal protein S23 expression and maximal protein synthesis *in vitro*. In COPD patients, miR-542-3p expression in quadriceps was elevated, and this may suppress physical performance, at least in part, by inhibiting mitochondrial and cytoplasmic ribosome synthesis and suppressing protein synthesis, leading to speculation that miR-542-3p may promote wasting (Farre-Garros et al., 2019). Also, miR-424-5p regulates rRNA synthesis by inhibiting Pol I pre-initiation complex formation, which reduces the capacity of the protein synthesis machinery, contributing to inhibition of protein synthesis and loss of muscle mass in patients with ICU-acquired weakness or COPD, patients undergoing aortic surgery, and in individuals from the Hertfordshire Sarcopenia Study (Connolly et al., 2018). miR-424-5p expression is elevated in muscle of cachectic non-small cell lung cancer (NSCLC) patients compared with healthy control subjects; however, the mechanism underlying the altered expression of miR-424-5p in NSCLC muscle atrophy is unknown (Worp et al., 2020). miR-142a-5p can inhibit mitofusin-1 (MFN1) expression by binding to the 3’UTR of its mRNA. Following overexpression of miR-142a-5p in C2C12 cells and a sciatic nerve transaction mouse model, MFN1 was downregulated, associated with extensive mitochondrial fragmentation, depolarization of mitochondrial membrane potential, accumulation of reactive oxygen species (ROS), and mitophagy and apoptosis activation in skeletal muscle cells, all of which aggravate muscle atrophy. Importantly, these effects were attenuated by overexpression of MFN1 (Yang et al., 2020).

### miRNAs That Attenuate Muscle Atrophy

Research strategies based on multiple muscular atrophy models and patient-derived muscle biopsies have also been applied to identify the miRNAs involved in decreasing muscle atrophy in cachexia. These studies have confirmed the crucial roles of miRNAs in regulating muscle wasting in cachexia, and miRNAs in different models of muscle wasting in cachexia can repress wasting via a variety of mechanisms.

**FIGURE 2:**
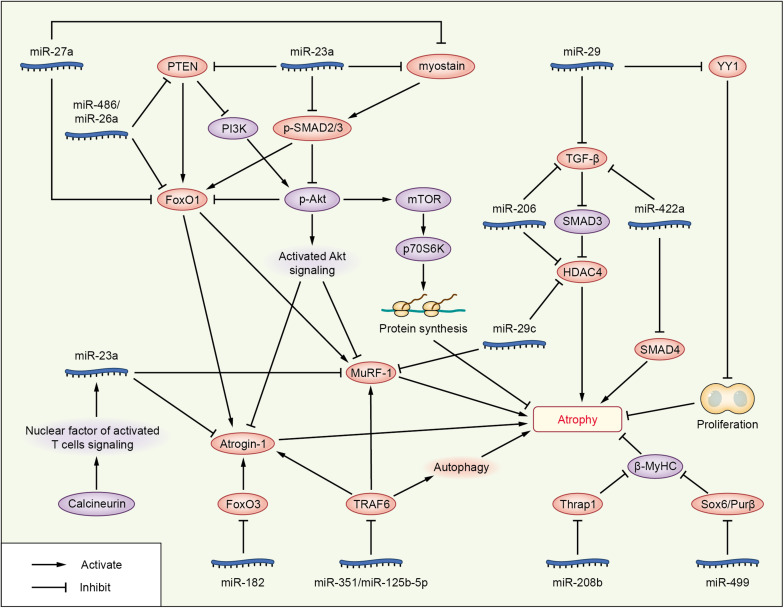
Roles of miRNAs in attenuating cachexia skeletal muscle atrophy. The miRNAs are represented in blue; molecules directly targeted by miRNAs are shown in pink; and other signaling molecules involved in muscle wasting are shown in purple.

Phosphatase and tensin homolog (PTEN), originally identified as a tumor suppressor, also plays a significant role in skeletal muscle atrophy (Wijesekara et al., 2005; Hu et al., 2007, 2010). It was reported that miRNAs attenuate muscle wasting of cachexia via PTEN/PI3K/Akt signaling (Figure 2 and Table 3). miR-486 and miR-26a repressed the translation of PTEN in a model of CKD, resulting in enhanced phosphorylation of Akt and FoxO1; miR-486 and miR-26a also directly inhibit the translation of FoxO1, and both of these effects lead to suppression of Atrogin-1 and MuRF-1 to prevent muscle atrophy (Small et al., 2010; Xu et al., 2012; Wang B. et al., 2019). miR-182 has also been reported to target FoxO. In muscle cells, miR-182 reduces FoxO3 expression and also blocks glucocorticoid-induced upregulation of multiple FoxO3 targets, including Atrogin-1, autophagy-related protein 12, cathepsin L, and microtubule-associated protein light chain 3, to decrease muscle wasting. miR-182 expression is decreased in C2C12 myotubes with Dex-induced muscle atrophy and in the gastrocnemius muscle of rats injected with streptozotocin-induced diabetes-related atrophy (Hudson et al., 2014a).

**TABLE 3:** Roles of miRNAs in attenuating skeletal muscle atrophy in cachexia and other diseases.

miRNAs	Partner	Model	References
miR-486 ↓	Phosphatase and tensin homolog (PTEN) ↑ and FoxO1 ↑	Chronic kidney disease (CKD) mice	Small et al., 2010; Xu et al., 2012
miR-26a ↓	FoxO1 ↑, PTEN ↑, and GSK-3β↑	CKD mice	Wang B. et al., 2019
miR-182 ↓	FoxO3 ↑	Diabetes mellitus rats and Dex-induced C2C12 myotubes	Hudson et al., 2014a
miR-23a/27a ↓	PTEN ↑, caspase-7 ↑, and FoxO1 ↑	CKD mice	Wang B. et al., 2017
	Atrogin-1 ↑, MuRF-1 ↑, and PTEN ↑	Diabetic mice	Zhang A. et al., 2018
miR-29 ↓	YY1↑ and TGF-β↑	Unilateral ureteral obstruction mice	Wang H. et al., 2019
miR-206 ↓	TGF-β↑	Denervation-induced rats	Huang et al., 2016
	HDAC4 ↑	Denervation-induced mice	Winbanks et al., 2013
miR-29c ↓	Atrogin-1↑, MuRF-1↑, and HDAC4 ↑	Mice and C2C12 myotubes	Silva et al., 2019
miR-23a ↓	Atrogin-1 ↑ and MuRF-1 ↑	Dex-induced mice and C2C12 myotubes	Wada et al., 2011
	Atrogin-1 ↑ and MuRF-1 ↑	Diabetic rats and Dex-induced C2C12 myotubes	Hudson et al., 2014b
miR-351 ↓	Tumor necrosis factor receptor –associated factor 6 (TRAF6) ↑	Denervation-induced rats and Dex-induced myotubes	He et al., 2016; Qiu et al., 2018
miR-125b-5p ↓	TRAF6 ↑	Denervated rats and fasting C2C12 myotubes	Qiu et al., 2019
miR-499 ↓	Sox6 ↑and Purβ↑	Hindlimb of rats	Panguluri et al., 2010
miR-208b ↓	Thrap1 ↑	Hindlimb of rats	Panguluri et al., 2010
miR-375 ↓	cyclin kinase cyclin D2 ↑, PAX6 ↑	Mouse and human neural stem cells	Bhinge et al., 2016
miR-2/miR-128 ↓	gar-2/m2R ↑	SMA models of C. elegans/mice	O’Hern et al., 2017
miR-23a ↓	/	iPSC-derived motor neurons of SMA patients; SMA mice	Kaifer et al., 2019
miR-206 ↓	sodium calcium exchanger isoform 2 ↑	SMA mice	Valsecchi et al., 2020

Overexpression of miR-23a/miR-27a in CKD mice enhanced grip strength, decreased muscle loss, increased the phosphorylation of Akt and FoxO1, decreased the activation of PTEN and FoxO1, and reduced Atrogin-1 and MuRF-1 protein levels, preventing muscle wasting via PTEN/PI3K/Akt signaling (Wang B. et al., 2017). These results are consistent with those of a streptozotocin-induced diabetic muscle atrophy model, and administration of miR-23a/27a weakened the diabetes-induced decrease in the muscle cross-sectional area and muscle function (Zhang A. et al., 2018). PTEN and caspase-7 are targets of miR-23a, and FoxO1 is a target of miR-27a, as identified by luciferase reporter assays in primary satellite cells (Wang B. et al., 2017). In a meta-analysis, miR-27a was showed as a new potential FoxO1 regulator during muscle wasting in cancer cachexia; miR-27a inhibits the expression of FoxO1, so as to inhibit muscle atrophy (Freire et al., 2019).

miR-23a/27a also participates in the myostatin or TGF-β/SMAD signaling pathways to decrease muscle atrophy, similar to other miRNAs, such as miR-29, miR-422a, and miR-206 (Figure 2 and Tables 2, 3). Administration of miR-23a/miR-27a also reduces myostatin expression and its downstream SMAD 2/3 signaling, diminishes caspase 3/7 activation, and increases the expression of muscle regeneration markers (Wang B. et al., 2017). In unilateral ureteral obstruction-induced muscle atrophy, miR-29 ameliorates atrophy and weakens renal fibrosis by suppressing the YY1 and TGF-β signaling proteins directly. YY1 inhibits muscle satellite cell proliferation, leading to the development of muscle wasting (Wang H. et al., 2019). In male patients with ICU-associated muscle weakness or COPD or who were undergoing aortic surgery, quadriceps expression of miR-422a was positively associated with muscle strength. In muscle cells *in vitro*, miR-422a targeted and restrained SMAD4 expression, a central component of the canonical TGF-β signaling pathway, and suppressed the luciferase activity induced by TGF-β and bone morphogenetic protein (Bmp). In addition, the preoperative miR-422a level is inversely correlated with muscle mass loss during the first week after surgery (Paul et al., 2018).

In a recent study, basic muscle characteristics, such as relative muscle weight, deteriorated continually during a 2-week period after surgical transection of the sciatic nerve. Injection of miR-206 attenuated the deterioration of muscle morphology and physiology, effectively blocked fibrosis, and suppressed TGF-β1 and HDAC4 expression as evaluated at 2 weeks after denervation. Moreover, miR-206 treatment increased the number of differentiating satellite cells, thereby protecting the denervated muscles from atrophy. Interestingly, the ability of miR-206 to regulate HDAC4 expression and to decrease muscle atrophy weakened after pharmacological blockade of the TGF-β/SMAD3 axis (Huang et al., 2016), suggesting that miR-206 may attenuate muscle atrophy via the TGF-β/SMAD3 axis. Other research on miR-206 showed that miR-206 targeted and inhibited HDAC4, and overexpression or inhibition of miR-206 in the muscles of mice reduced or increased HDAC4 levels, respectively, but did not alter muscle mass or myofiber size. Vector-mediated manipulation of miR-206 activity in follistatin-induced hypertrophy and denervation-induced atrophy models did not alter the gain and loss of muscle mass, respectively; miR-206 repressed the hypertrophy of myogenic cells, but not muscle fibers via inhibition of HDAC4 (Winbanks et al., 2013). These two studies showing that miR-206 decreased muscle atrophy are in contrast to another study reporting that miR-206 promotes wasting (Soares et al., 2014). HDAC4 is also targeted by miR-29c, which decreases muscle atrophy (Silva et al., 2019).

In addition to targeting HDAC4, miR-29c may reduce muscle wasting via other pathways (Figure 2 and Table 3). miR-29c can bind to the 3’UTR of MuRF-1 to inhibit its expression. miR-29c is reported to improve skeletal muscle size and function by stimulating satellite cell proliferation and suppressing atrophy-related genes. Overexpression of miR-29c inhibits the expression of Atrogin-1, MuRF-1, and HDAC4 (Silva et al., 2019). miR-23a can also inhibit the translation of both Atrogin-1 and MuRF-1 in a 3’UTR-dependent manner. Ectopic expression of miR-23a is sufficient to protect muscles from Dex-induced atrophy *in vitro* and *in vivo* (Wada et al., 2011). In another study, miR-23a was decreased in the gastrocnemius of rats with acute streptozotocin-induced diabetes, a condition known to increase the expression of Atrogin-1 and MuRF-1 and to cause atrophy; miR-23a also reduced in a Dex-induced atrophy model of C2C12 myotubes. The two atrophy-inducing conditions downregulated miR-23a in muscles by mechanisms involving attenuation of the calcineurin/nuclear factor of activated T cells signaling pathway (Hudson et al., 2014b). miR-351 can inhibit denervation-induced atrophy of tibialis anterior (TA) muscles following sciatic nerve transection, at least partially, via negative regulation of tumor necrosis factor receptor-associated factor 6 (TRAF6), as well as Atrogin-1 and MuRF-1, two signaling molecules downstream of TRAF6 (He et al., 2016). This is consistent with the results of the Dex-induced myotube atrophy model (Qiu et al., 2018). TRAF6 is also directly targeted by miR-125b-5p. The expression of miR-125b-5p is downregulated in both atrophic fasting C2C12 myotubes and denervated TA muscles (Qiu et al., 2019), and our research showed that several miRNAs were downregulated under atrophic fasting/starvation conditions in C2C12 myotubes or mice (Lei et al., 2019). Overexpression of miR-125b-5p protects skeletal muscle samples by targeting TRAF6 via downregulation of Atrogin-1, MuRF-1, and autophagy-lysosome system-related proteins (Qiu et al., 2019).

In addition to the above signaling pathway, some miRNAs can decrease muscle atrophy by regulating other molecules (Figure 2 and Table 3). Hindlimb suspension decreases expression of miR-221, miR-499, and miR-208b, increases the levels of Sox6, Purβ (miR-499 targets), and Thrap1 (miR-208b target). Sox6, Purβ, and Thrap1 can decrease β-MyHC expression in rat soleus muscle during skeletal muscle atrophy (McCarthy et al., 2009), suggesting that miR-221, miR-499, and miR-208b may inhibit muscle wasting. Whereas TNF-like weak inducer of apoptosis (TWEAK)-induced skeletal muscle wasting of C2C12 myotubes caused downregulation of miR-1, miR-133a, miR-133b, miR-206, miR-27, miR-23, miR-93, miR-199, miR-107, and miR-192, TWEAK increased the expression of miR-715, miR-146a, miR-455, miR-322, miR-98, and miR-470 in C2C12 myotubes via an unknown mechanism (Panguluri et al., 2010).

miRNAs also play important roles in reducing the pathology of spinal muscular atrophy (SMA) (Table 3). SMA is an autosomal recessive neuromuscular disease, which is caused by deletions or mutations in the survival motor neuron (SMN) gene (Ross and Kwon, 2019). miR-375 promote spinal motor neurogenesis by targeting the cyclin kinase cyclin D2 and the transcription factor Pax6 in mouse and human neural stem cells. Besides, miR-375 inhibits p53 and protects neurons from apoptosis in response to DNA damage, suggesting that miR-375 may play a protective role in motor neurons (Bhinge et al., 2016). In SMA models of C. elegans/mice, miR-2/miR-128 targets gar-2/m2R mRNA in cholinergic neurons and miR-2/miR-128 is required for neuromuscular junction function. miR-2 binds and inhibits *gar-2* mRNA translation, but does not reduce transcript levels; and gar-2 loss ameliorates smn-1 neuromuscular defects, suggesting that miR-2/miR-128 may ameliorate neuromuscular defects (O’Hern et al., 2017). In SMA patients and mice, miR-23a significantly reduces the pathology in SMA mice, including increased motor neuron size, reduced neuromuscular junction pathology, increased muscle fiber area, and extended survival (Kaifer et al., 2019). miR-206 reduces the severity of SMA pathology, slowing down disease progression, improving behavioral performance and increasing survival rate of mice. miRNA-206 upregulation induces a reduction of the predicted target sodium calcium exchanger isoform 2, one of the main regulators of intracellular [Ca^2+^] and [Na^+^] (Valsecchi et al., 2020).

These data validate the roles of miRNAs in mediating the effects of anabolic and catabolic factors. Especially, experimentally manipulated miRNA expression can attenuate or even reverse skeletal muscle atrophy during cachexia and other diseases, suggesting the substantial contribution of miRNAs in regulating skeletal muscle mass and functions in muscle wasting.

## lncRNAs Involved in Skeletal Muscle Atrophy

**FIGURE 3:**
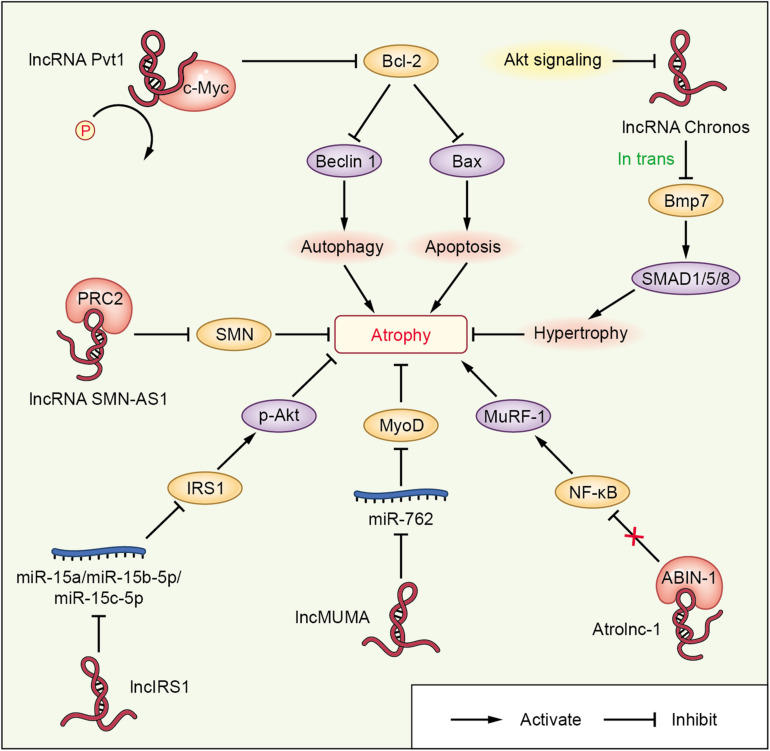
lncRNA-mediated networks regulating skeletal muscle atrophy in cachexia and other diseases. The lncRNAs are represented in red; miRNAs or proteins directly targeted by lncRNAs are shown in blue or pink, respectively; the molecules regulated downstream of miRNAs or proteins interacting with lncRNAs are shown in orange; and other signaling molecules involved in muscle wasting are shown in purple.

lncRNAs are ncRNAs longer than 200 nucleotides. A small group of lncRNAs are reportedly expressed in muscle, and they have emerged as significant regulators that promote or attenuate muscle atrophy in cachexia and other diseases (Figure 3 and Table 4). These lncRNAs include the lncRNAs Chronos, atrophy-related long non-coding RNA-1 (Atrolnc-1), mechanical unloading-induced muscle atrophy-related lncRNA (lncMUMA), and lncIRS1 (TCONS-00086268). Studies on the regulation of muscle atrophy in cachexia by lncRNAs are just in the infant stage, the descriptions of lncRNA Pvt1 and SMN-AS1 in other diseases is helpful to a more comprehensive understanding of the relationship between lncRNA and muscle atrophy.

**TABLE 4:** lncRNA-mediated networks regulating skeletal muscle atrophy in cachexia and other diseases.

lncRNAs	Roles and mechanisms in regulating skeletal muscle wasting	Model	References
lncRNA Chronos (lncRNA Gm17281) ↑	Akt signaling ↓ – lncRNA Chronos ↑ – Bmp7 transcription in *trans*↓ – promoting muscular wasting	Mice (aged, hind limb unloading, type 1 diabetes)	Neppl et al., 2017
Atrophy-related long non-coding RNA-1 (Atrolnc-1) ↑	Interacting with ABIN-1 – activating NF-κB – MuRF-1 ↑ – leading to muscle wasting	Mice (CKD, starvation, cancer)	Sun et al., 2018
Mechanical unloading- induced muscle atrophy-related lncRNA (lncMUMA) ↑	Sponging miR-762 – MyoD ↑ – myogenic differentiation ↑ – blocking muscle wasting	Hindlimb suspension mice and microgravity-simulated C2C12 myoblasts	Zhang et al., 2018a
lncIRS1 (TCONS_00086268) ↑	Sponging miR-15 family – IRS1 ↑ – activating IGF-1/PI3K/Akt pathway – rescuing atrophy	Dex-treated chicken DF-1 cells	Li et al., 2019
lncRNA Pvt1 ↑	Preventing c-Myc degradation – Bcl-2 ↓ – autophagy and apoptosis ↑ – leading to muscle wasting	Mice (amyotrophic lateral sclerosis, denervation)	Alessio et al., 2019
lncRNA SMN-AS1 ↑	Recruiting PRC2 to the survival motor neuron (SMN) promoter – SMN transcription ↓ – increases muscle wasting	Spinal muscular atrophy mice	Woo et al., 2017

lncRNA Chronos (lncRNA Gm17281), located on chromosome 2, is a muscle-enriched, aging-related lncRNA that is inhibited by Akt and serves as an inhibitor of hypertrophic growth (Neppl et al., 2017), suggesting that Chronos may lead to muscular wasting by restraining hypertrophic growth. Chronos, however, does not appear to function as a classical ‘atrogene’ because its expression is uncoupled from the loss of muscle mass in atrophy models of aging, hind limb unloading, and streptozotocin-induced type 1 diabetes mice. Chronos is negatively regulated by Akt signaling and positively regulated by aging. Chronos can repress hypertrophic growth *in vitro* and *in vivo*, in part, by in *trans* negatively regulating Bmp7 expression, which is located on chromosome 20 (Neppl et al., 2017). This is consistent with prior studies reporting hypertrophic effects of Bmp signaling, which has been shown to positively regulate skeletal muscle hypertrophy via activation of SMAD1/5/8 (Sartori et al., 2013).

Atrolnc-1 increases muscle wasting in fasting mice and mice with cancer, or chronic kidney disease (CKD). Reduced insulin signaling promotes the binding of the transcription factor C/EBP-α to the Atrolnc-1 promoter and increases Atrolnc-1 expression. In cultured C2C12 myotubes, overexpression of Atrolnc-1 promotes protein degradation, whereas Atrolnc-1 knockdown significantly decreases the protein degradation rate increased by serum depletion. Atrolnc-1 interacts with A20 binding inhibitor of NF-κB-1 to inhibit its function, leading to heightened NF-κB activity and MuRF-1 transcription. In the TA muscle of normal mice, overexpression of Atrolnc-1 increases MuRF-1 expression, which results in a loss of myofibers. Knockdown of Atrolnc-1 reduced muscle atrophy in mice with CKD by inhibiting NF-κB activity and MuRF-1 expression (Sun et al., 2018).

In addition to increasing muscle wasting, lncRNAs have also been reported to attenuate muscle atrophy. Mechanical unloading-induced muscle atrophy-related lncRNA (lncMUMA), which is enriched in muscles, can reverse the muscle atrophy induced by hindlimb suspension. lncMUMA is the most downregulated lncRNA during muscle wasting induced by hindlimb suspension mice. *In vitro* and *in vivo* data show that the reduction in lncMUMA is closely related to decreased myogenesis during mechanical unloading. *In vitro*, lncMUMA functions as a miR-762 sponge and facilitates myogenic differentiation by modulating MyoD. Overexpression of lncMUMA alleviates the loss of MyoD protein and muscle mass in miR-762 knock-in mice. In microgravity simulation, overexpression of lncMUMA can accelerate the myogenic differentiation of myoblasts and block the development of muscle wasting *in vitro* (Zhang et al., 2018a).

lncIRS1 can regulate the expression of atrophy-related genes and rescue muscle wasting. lncIRS1 is particularly enriched in skeletal muscle. lncIRS1 can regulate proliferation and differentiation of myoblasts *in vitro* and the mass and cross-sectional area of muscle fibers *in vivo*. The expression of lncIRS1 gradually increases with myogenic differentiation. lncIRS1 can act as a ceRNA for miR-15a, miR-15b-5p, and miR-15c-5p to modulate the expression of IRS1, which is downstream of the IGF-1 receptor. Overexpression of lncIRS1 accelerates the protein expression of IRS1 and facilitates p-Akt signaling, which is a core component of the IGF-1 signaling pathway, leading to the promotion, proliferation, and differentiation of myoblasts and the rescue of muscle atrophy (Li et al., 2019).

Single cell analysis showed that the lncRNA Pvt1, which is activated in early muscle wasting, is involved in promoting muscle atrophy and modulating the mitochondrial network. Pvt1 silencing in models of denervation and amyotrophic lateral sclerosis atrophy resulted in a less fragmented mitochondrial network compared with the controls (Alessio et al., 2019). Pvt1 can prevent c-Myc degradation by hindering phosphorylation of Thr58 in c-Myc (Johnsson and Morris, 2014). c-Myc inhibits the anti-apoptotic protein Bcl-2 (Eischen et al., 2001), which is a key promoter of apoptosis and autophagy, two vital events that occur during muscle atrophy. Bcl-2 can inhibit Beclin 1 and Bax, which enhance autophagy and apoptosis, respectively, leading to muscle wasting (Alessio et al., 2019).

lncRNAs, such as SMN-AS1, also play important roles in accelerating muscle atrophy in SMA. lncRNA SMN-AS1 is transcribed from the antisense strand of the SMN gene and is highly enriched in neurons (D Ydewalle et al., 2017). SMN-AS1 increases muscle wasting by recruiting polycomb repressive complex 2 (PRC2) to the SMN promoter and restricting SMN transcription (Woo et al., 2017). In addition, selective destruction of SMN-AS1-mediated PRC2 recruitment results in activated SMN, attenuated muscle atrophy, and improved SMA phenotypes in mice (Woo et al., 2017).

Recently, several studies have demonstrated the crucial roles of lncRNAs in regulating skeletal muscle atrophy. lncRNAs have been identified as metabolic regulators promoting or reversing muscle atrophy in a variety of wasting models; however, our comprehension of the biological functions of lncRNAs in wasting is still in its infancy. Knowledge of the mechanisms of lncRNA regulation of muscle atrophy may aid the development of potential therapeutics for the clinical treatment of muscle atrophy in cachexia and other diseases.

## Conclusion and Perspectives

Skeletal muscle, which can contract or stretch, is vital for body metabolism, homeostasis, and movement (Agudelo et al., 2019; Amoasii et al., 2019; Fiorenza et al., 2019). The development of skeletal muscle has been studied in depth, and a variety of regulatory molecules and complex mechanisms are involved in skeletal muscle development. However, when the normal development of skeletal muscle is disrupted, pathological conditions develop.

Skeletal muscle atrophy in cachexia is due to disruption of the balance between protein synthesis and degradation, which can be partially, but not entirely, reversed by nutritional support (Baracos et al., 2018; de Castro et al., 2019). Weight loss is a significant feature of cachexia and is closely associated with mortality (de Castro et al., 2019). Cachexia is very common in cancer patients, with up to 80% of patients having advanced-stage disease (Porporato, 2016), and cachexia is the immediate cause of death in at least 20% of all cancer patients (Porporato, 2016). Interventions to block or attenuate muscle wasting, while preventing the progression of cachexia, are highly desirable.

The discovery of ncRNAs has opened up a new chapter in medicine (Adams et al., 2017; Rupaimoole and Slack, 2017; Levin, 2019), of which we have just begun to scratch the surface. Focusing on disease-specific and tissue-specific ncRNAs seems to be an ideal approach to identify candidate biomarkers or therapeutic targets, for example, lncRNA PCA3 (also known as DD3) in prostate cancer (Hessels et al., 2003; Tomlins et al., 2011, 2016; Mitra et al., 2020). lncRNA PCA3 is the first ncRNA to receive Food and Drug Administration approval as a cancer biomarker test (Hessels et al., 2003). Although therapeutic applications are still in their infancy, drugs targeting ncRNAs that participate in cancer signaling will hopefully be used to treat cancer patients one day.

In addition to the regulations and potential applications of ncRNAs in cancers, increasing research on the functions of ncRNAs in skeletal muscle suggests that miRNAs and lncRNAs are significant regulators of muscular atrophy in cachexia and other diseases (Crone et al., 2012; Alexander et al., 2014; Narasimhan et al., 2017; Cao et al., 2018; Okugawa et al., 2018; Mitra et al., 2020; Shuai et al., 2020). The roles of miRNAs in skeletal muscle in certain diseases have been well described in different muscular atrophy models, and in recent years, the involvement of miRNAs in regulating muscular atrophy in cachexia has been confirmed. These miRNAs could serve not only as biomarkers for muscle status and wasting but also as biomarkers to monitor muscle regeneration and therapy effects (Suzuki and Springer, 2018). miRNAs associated with muscular atrophy in disease are expected to be identified in the future, and they may function by many different mechanisms. In addition, lncRNAs are reported to participate in various physiological and pathological processes, such as cancer (Lu et al., 2013; He et al., 2014; Sun et al., 2014; Zhuang et al., 2014; Yin et al., 2015; Zhou et al., 2015; Zou et al., 2016; Wang J. et al., 2017), aging (Boon et al., 2016; Su et al., 2019), muscle development (Ballarino et al., 2018; Jin et al., 2018; Zhang et al., 2018b), and their associated disorders (Han et al., 2020). A better understanding of the roles of miRNAs and lncRNAs and their corresponding signaling pathways in regulating skeletal muscle atrophy in cachexia and other diseases will eventually help elucidate the mechanisms underlying muscle wasting and may contribute to the design of new strategies for the prevention, diagnosis, and treatment of muscle-wasting diseases.

In summary, there have been significant advances in our understanding of the crucial roles of miRNAs and lncRNAs in regulating skeletal muscle development (Cesana et al., 2011; Legnini et al., 2014; Zhu et al., 2017; Kong et al., 2019; Cheng et al., 2020; Li et al., 2020). A limited number of studies have specifically identified roles of miRNAs and lncRNAs in muscular atrophy in cachexia and other diseases, and these studies suggest that miRNAs and lncRNAs are central to abnormal regulation of protein synthesis and degradation. Moreover, preliminary evidence suggests that miRNAs and lncRNAs are involved in muscle-organ crosstalk that leads to muscle disorder in COPD (Paul et al., 2018), CKD (Wang B. et al., 2019; Zhang et al., 2019), and heart failure (Kumarswamy et al., 2014; Santer et al., 2019). The roles of altered miRNA (such as miR-29b) and lncRNA levels warrant further investigation in muscle wasting and cachexia (Bei and Xiao, 2017). This phenomenon may hold great potential for the development of possible strategies to treat muscle, pulmonary, kidney, and heart diseases contemporaneously. The challenge is how to use these miRNAs and lncRNAs to develop more effective, economic, preventive, diagnostic, and therapeutic tools to improve the quality of life and decrease the mortality of patients with muscular atrophy in cachexia and other diseases.

## Author Contributions

RC conceived the study. RC and SL drafted and revised the manuscript. TJ, YS, and HS performed the literature search and contributed to the draft. All authors read and approved the final version.

## Conflict of Interest

The authors declare that the research was conducted in the absence of any commercial or financial relationships that could be construed as a potential conflict of interest.

## References

[B1] Adams B. D. Parsons C. Walker L. Zhang W. C. Slack F. J. (2017). Targeting noncoding RNAs in disease. *J. Clin. Invest.* 127 761–771. 10.1172/JCI84424 28248199 PMC5330746

[B2] Agudelo L. Z. Ferreira D. M. S. Dadvar S. Cervenka I. Ketscher L. Izadi M. (2019). Skeletal muscle PGC-1α1 reroutes kynurenine metabolism to increase energy efficiency and fatigue-resistance. *Nat. Commun.* 10:10712. 10.1038/s41467-019-10712-0 31235694 PMC6591322

[B3] Alessio E. Buson L. Chemello F. Peggion C. Grespi F. Martini P. (2019). Single cell analysis reveals the involvement of the long non-coding RNA Pvt1 in the modulation of muscle atrophy and mitochondrial network. *Nucleic Acids Res.* 47 1653–1670. 10.1093/nar/gkz007 30649422 PMC6393313

[B4] Alexander M. S. Casar J. C. Motohashi N. Vieira N. M. Eisenberg I. Marshall J. L. (2014). MicroRNA-486–dependent modulation of DOCK3/PTEN/AKT signaling pathways improves muscular dystrophy–associated symptoms. *J. Clin. Invest.* 124 2651–2667. 10.1172/JCI73579 24789910 PMC4038577

[B5] Amoasii L. Sanchez-Ortiz E. Fujikawa T. Elmquist J. K. Bassel-Duby R. Olson E. N. (2019). NURR1 activation in skeletal muscle controls systemic energy homeostasis. *Proc. Natl. Acad. Sci. U.S.A.* 116 11299–11308. 10.1073/pnas.1902490116 31110021 PMC6561277

[B6] Anker S. D. Ponikowski P. Varney S. Chua T. P. Clark A. L. Webb-Peploe K. M. (1997). Wasting as independent risk factor for mortality in chronic heart failure. *Lancet* 349 1050–1053. 10.1016/S0140-6736(96)07015-8 9107242

[B7] Ballarino M. Cipriano A. Tita R. Santini T. Desideri F. Morlando M. (2018). Deficiency in the nuclear long noncoding RNA Charme causes myogenic defects and heart remodeling in mice. *Embo J.* 37:e99697. 10.15252/embj.201899697 30177572 PMC6138438

[B8] Baracos V. E. Martin L. Korc M. Guttridge D. C. Fearon K. C. H. (2018). Cancer-associated cachexia. *Nat. Rev. Dis. Primers* 4:105. 10.1038/nrdp.2017.105 29345251

[B9] Bei Y. Xiao J. (2017). MicroRNAs in muscle wasting and cachexia induced by heart failure. *Nat. Rev. Cardiol.* 14:566. 10.1038/nrcardio.2017.122 28770869

[B10] Bhinge A. Namboori S. C. Bithell A. Soldati C. Buckley N. J. Stanton L. W. (2016). MiR-375 is essential for human spinal motor neuron development and may be involved in motor neuron degeneration. *Stem Cells* 34 124–134. 10.1002/stem.2233 26507573

[B11] Blattler S. M. Verdeguer F. Liesa M. Cunningham J. T. Vogel R. O. Chim H. (2012). Defective mitochondrial morphology and bioenergetic function in mice lacking the transcription factor yin yang 1 in skeletal muscle. *Mol. Cell Biol.* 32 3333–3346. 10.1128/MCB.00337-12 22711985 PMC3434543

[B12] Boon R. A. Hofman P. Michalik K. M. Lozano-Vidal N. Berghäuser D. Fischer A. (2016). Long noncoding RNA Meg3 controls endothelial cell aging and function. *J. Am. Coll Cardiol.* 16 2589–2591. 10.1016/j.jacc.2016.09.949 27931619

[B13] Cao L. Liu Y. Wang D. Huang L. Li F. Liu J. (2018). MiR-760 suppresses human colorectal cancer growth by targeting BATF3/AP-1/cyclinD1 signaling. *J. Exp. Clin. Canc. Res.* 37:83. 10.1186/s13046-018-0757-8 29661228 PMC5902951

[B14] Cesana M. Cacchiarelli D. Legnini I. Santini T. Sthandier O. Chinappi M. (2011). A long noncoding RNA controls muscle differentiation by functioning as a competing endogenous RNA. *Cell* 147 358–369. 10.1016/j.cell.2011.09.028 22000014 PMC3234495

[B15] Chen J. Mandel E. M. Thomson J. M. Wu Q. Callis T. E. Hammond S. M. (2006). The role of microRNA-1 and microRNA-133 in skeletal muscle proliferation and differentiation. *Nat. Genet.* 38 228–233. 10.1038/ng1725 16380711 PMC2538576

[B16] Chen J. Tao Y. Li J. Deng Z. Yan Z. Xiao X. (2010). microRNA-1 and microRNA-206 regulate skeletal muscle satellite cell proliferation and differentiation by repressing Pax7. *J. Cell Biol.* 190 867–879. 10.1083/jcb.200911036 20819939 PMC2935565

[B17] Chen R. Jiang T. She Y. Xie S. Zhou S. Li C. (2018). Comprehensive analysis of lncRNAs and mRNAs with associated co-expression and ceRNA networks in C2C12 myoblasts and myotubes. *Gene* 647 164–173. 10.1016/j.gene.2018.01.039 29331478

[B18] Chen R. Jiang T. She Y. Xu J. Li C. Zhou S. (2017). Effects of cobalt chloride, a hypoxia-mimetic agent, on autophagy and atrophy in skeletal C2C12 myotubes. *Biomed. Res. Int.* 2017 1–9. 10.1155/2017/7097580 28706950 PMC5494548

[B19] Chen R. Lei S. Jiang T. Zeng J. Zhou S. She Y. (2020). Roles of lncRNAs and circRNAs in regulating skeletal muscle development. *Acta Physiol. (Oxf.)* 228:e13356. 10.1111/apha.13356 31365949

[B20] Chen R. She Y. Fu Q. Chen X. Shi H. Lei S. (2019). Differentially expressed coding and noncoding RNAs in CoCl2–induced cytotoxicity of C2C12 cells. *Epigenomics UK* 11 423–438. 10.2217/epi-2018-0087 30785338

[B21] Cheng X. Li L. Shi G. Chen L. Fang C. Li M. (2020). MEG3 promotes differentiation of porcine satellite cells by sponging miR-423-5p to relieve inhibiting effect on SRF. *Cells Basel* 9:449. 10.3390/cells9020449 32075310 PMC7072828

[B22] Cichewicz M. A. Kiran M. Przanowska R. K. Sobierajska E. Shibata Y. Dutta A. (2018). MUNC, an enhancer RNA upstream from the MYOD gene, induces a subgroup of myogenic transcripts in trans independently of MyoD. *Mol. Cell Biol.* 38:e00655-17. 10.1128/MCB.00655-17 30037979 PMC6168980

[B23] Connolly M. Paul R. Farre-Garros R. Natanek S. A. Bloch S. Lee J. (2018). miR-424-5p reduces ribosomal RNA and protein synthesis in muscle wasting. *J. Cachex. Sarcop. Muscle* 9 400–416. 10.1002/jcsm.12266 29215200 PMC5879973

[B24] Crone S. G. Jacobsen A. Federspiel B. Bardram L. Krogh A. Lund A. H. (2012). microRNA-146a inhibits G protein-coupled receptor-mediated activation of NF-κB by targeting CARD10 and COPS8 in gastric cancer. *Mol. Cancer* 11:71. 10.1186/1476-4598-11-71 22992343 PMC3515505

[B25] de Castro G. S. Simoes E. Lima J. D. C. C. Ortiz-Silva M. Festuccia W. T. Tokeshi F. (2019). Human cachexia induces changes in mitochondria, autophagy and apoptosis in the skeletal muscle. *Cancers* 11:1264. 10.3390/cancers11091264 31466311 PMC6770124

[B26] Eischen C. M. Packham G. Nip J. Fee B. E. Hiebert S. W. Zambetti G. P. (2001). Bcl-2 is an apoptotic target suppressed by both c-Myc and E2F-1. *Oncogene* 20 6983–6993. 10.1038/sj.onc.1204892 11704823

[B27] Faghihi M. A. Modarresi F. Khalil A. M. Wood D. E. Sahagan B. G. Morgan T. E. (2008). Expression of a noncoding RNA is elevated in Alzheimer’s disease and drives rapid feed-forward regulation of β-secretase. *Nat. Med.* 14 723–730. 10.1038/nm1784 18587408 PMC2826895

[B28] Farre-Garros R. Lee J. Y. Natanek S. A. Connolly M. Sayer A. A. Patel H. (2019). Quadriceps miR-542-3p and -5p are elevated in COPD and reduce function by inhibiting ribosomal and protein synthesis. *J. Appl. Physiol.* 126 1514–1524. 10.1152/japplphysiol.00882.2018 30676868 PMC6551227

[B29] Fearon K. Strasser F. Anker S. D. Bosaeus I. Bruera E. Fainsinger R. L. (2011). Definition and classification of cancer cachexia: an international consensus. *Lancet Oncol.* 12 489–495. 10.1016/S1470-2045(10)70218-7 21296615

[B30] Fiorenza M. Lemminger A. K. Marker M. Eibye K. Marcello Iaia F. Bangsbo J. (2019). High-intensity exercise training enhances mitochondrial oxidative phosphorylation efficiency in a temperature–dependent manner in human skeletal muscle: implications for exercise performance. *FASEB J.* 33 8976–8989. 10.1096/fj.201900106RRR 31136218

[B31] Freire P. P. Fernandez G. J. Cury S. S. de Moraes D. Oliveira J. S. de Oliveira G. (2019). The pathway to cancer cachexia: microRNA-regulated networks in muscle wasting based on integrative meta-analysis. *Int. J. Mol. Sci.* 20:1962. 10.3390/ijms20081962 31013615 PMC6515458

[B32] Garros R. F. Paul R. Connolly M. Lewis A. Garfield B. E. Natanek S. A. (2017). MicroRNA-542 promotes mitochondrial dysfunction and SMAD activity and is elevated in intensive care unit–acquired weakness. *Am. J. Resp. Crit. Care* 196 1422–1433. 10.1164/rccm.201701-0101OC 28809518 PMC5736972

[B33] Han Y. Liu Y. Yang C. Gao C. Guo X. Cheng J. (2020). LncRNA CASC2 inhibits hypoxia-induced pulmonary artery smooth muscle cell proliferation and migration by regulating the miR-222/ING5 axis. *Cell Mol. Biol. Lett.* 25:215. 10.1186/s11658-020-00215-y 32206065 PMC7079380

[B34] He Q. Qiu J. Dai M. Fang Q. Sun X. Gong Y. (2016). MicroRNA-351 inhibits denervation-induced muscle atrophy by targeting TRAF6. *Exp. Ther. Med.* 12 4029–4034. 10.3892/etm.2016.3856 28101181 PMC5228305

[B35] He Y. Wu Y. Huang C. Meng X. Ma T. Wu B. (2014). Inhibitory effects of long noncoding RNA MEG3 on hepatic stellate cells activation and liver fibrogenesis. *Biochim. Biophys. Acta (BBA) Mol. Basis Dis.* 1842 2204–2215. 10.1016/j.bbadis.2014.08.015 25201080

[B36] Hessels D. Klein Gunnewiek J. M. T. van Oort I. Karthaus H. F. M. van Leenders G. J. L. van Balken B. (2003). DD3PCA3-based molecular urine analysis for the diagnosis of prostate cancer. *Eur. Urol.* 44 8–16. 10.1016/S0302-2838(03)00201-X 12814669

[B37] Hu Z. Lee I. H. Wang X. Sheng H. Zhang L. Du J. (2007). PTEN expression contributes to the regulation of muscle protein degradation in diabetes. *Diabetes* 56 2449–2456. 10.2337/db06-1731 17623817

[B38] Hu Z. Wang H. Lee I. H. Modi S. Wang X. Du J. (2010). PTEN inhibition improves muscle regeneration in mice fed a high-fat diet. *Diabetes* 59 1312–1320. 10.2337/db09-1155 20200318 PMC2874691

[B39] Huang Q. K. Qiao H. Fu M. Li G. Li W. Chen Z. (2016). MiR-206 attenuates denervation-induced skeletal muscle atrophy in rats through regulation of satellite cell differentiation via TGF-β1, Smad3, and HDAC4 signaling. *Med. Sci. Monitor.* 22 1161–1170. 10.12659/MSM.897909 27054781 PMC4829125

[B40] Hudson M. B. Rahnert J. A. Zheng B. Woodworth-Hobbs M. E. Franch H. A. Price S. R. (2014a). miR-182 attenuates atrophy-related gene expression by targeting FoxO3 in skeletal muscle. *Am. J. Physiol. Cell Physiol.* 307 C314–C319. 10.1152/ajpcell.00395.2013 24871856 PMC4137139

[B41] Hudson M. B. Woodworth-Hobbs M. E. Zheng B. Rahnert J. A. Blount M. A. Gooch J. L. (2014b). miR-23a is decreased during muscle atrophy by a mechanism that includes calcineurin signaling and exosome-mediated export. *Am. J. Physiol. Cell Physiol.* 306 C551–C558. 10.1152/ajpcell.00266.2013 24336651 PMC3948973

[B42] Iannone F. Montesanto A. Cione E. Crocco P. Caroleo M. C. Dato S. (2020). Expression patterns of muscle-specific miR-133b and miR-206 correlate with nutritional status and sarcopenia. *Nutrients* 12:297. 10.3390/nu12020297 31979011 PMC7071413

[B43] Jin J. J. Lv W. Xia P. Xu Z. Y. Zheng A. D. Wang X. J. (2018). Long noncoding RNA SYISL regulates myogenesis by interacting with polycomb repressive complex 2. *Proc. Natl. Acad. Sci. U.S.A.* 115 E9802–E9811. 10.1073/pnas.1801471115 30279181 PMC6196504

[B44] Johnsson P. Morris K. V. (2014). Expanding the functional role of long noncoding RNAs. *Cell Res.* 24 1284–1285. 10.1038/cr.2014.104 25104732 PMC4220152

[B45] Kaifer K. A. Villalón E. O’Brien B. S. Sison S. L. Smith C. E. Simon M. E. (2019). AAV9-mediated delivery of miR-23a reduces disease severity in Smn^2B/–^SMA model mice. *Hum. Mol. Genet.* 28 3199–3210. 10.1093/hmg/ddz142 31211843 PMC6859438

[B46] Kong D. He M. Yang L. Zhou R. Yan Y. Q. Liang Y. (2019). MiR-17 and miR-19 cooperatively promote skeletal muscle cell differentiation. *Cell Mol. Life Sci.* 76 5041–5054. 10.1007/s00018-019-03165-7 31214725 PMC6881278

[B47] Kukreti H. Amuthavalli K. Harikumar A. Sathiyamoorthy S. Feng P. Z. Anantharaj R. (2013). Muscle-specific MicroRNA1 (miR1) targets heat shock protein 70 (HSP70) during dexamethasone-mediated atrophy. *J. Biol. Chem.* 288 6663–6678. 10.1074/jbc.M112.390369 23297411 PMC3585106

[B48] Kumarswamy R. Bauters C. Volkmann I. Maury F. Fetisch J. Holzmann A. (2014). Circulating long noncoding RNA, LIPCAR, predicts survival in patients with heart failure. *Circ Res.* 114 1569–1575. 10.1161/CIRCRESAHA.114.303915 24663402

[B49] Legnini I. Morlando M. Mangiavacchi A. Fatica A. Bozzoni I. (2014). A feedforward regulatory loop between HuR and the long noncoding RNA linc-MD1 controls early phases of myogenesis. *Mol. Cell* 53 506–514. 10.1016/j.molcel.2013.12.012 24440503 PMC3919156

[B50] Lei S. She Y. Zeng J. Chen R. Zhou S. Shi H. (2019). Expression patterns of regulatory lncRNAs and miRNAs in muscular atrophy models induced by starvation in vitro and in vivo. *Mol. Med. Rep.* 20 4175–4185. 10.3892/mmr.2019.10661 31545487 PMC6798001

[B51] Levin A. A. (2019). Treating disease at the RNA level with oligonucleotides. *New Engl. J. Med.* 380 57–70. 10.1056/NEJMra1705346 30601736

[B52] Li J. Chan M. C. Yu Y. Bei Y. Chen P. Zhou Q. (2017). miR-29b contributes to multiple types of muscle atrophy. *Nat. Commun.* 8:15201. 10.1038/ncomms15201 28541289 PMC5458521

[B53] Li J. Wang L. Hua X. Tang H. Chen R. Yang T. (2020). CRISPR/Cas9-mediated miR-29b editing as a treatment of different types of muscle atrophy in mice. *Mol. Ther.* 28:5. 10.1016/j.ymthe.2020.03.005 32222157 PMC7210721

[B54] Li Z. Cai B. Abdalla B. A. Zhu X. Zheng M. Han P. (2019). LncIRS1 controls muscle atrophy via sponging miR - 15 family to activate IGF1–PI3K/AKT pathway. *J. Cachex. Sarcop. Muscle* 10 391–410. 10.1002/jcsm.12374 30701698 PMC6463472

[B55] Liu C. Wang M. Chen M. Zhang K. Gu L. Li Q. (2017). miR-18a induces myotubes atrophy by down-regulating IgfI. *Int. J. Biochem. Cell Biol.* 90 145–154. 10.1016/j.biocel.2017.07.020 28782600

[B56] Lok C. (2015). Cachexia: the last illness. *Nature* 528 182–183. 10.1038/528182a 26659165

[B57] Lu K. Li W. Liu X. Sun M. Zhang M. Wu W. (2013). Long non-coding RNA MEG3 inhibits NSCLC cells proliferation and induces apoptosis by affecting p53 expression. *BMC Cancer* 13:461. 10.1186/1471-2407-13-461 24098911 PMC3851462

[B58] Lu L. Sun K. Chen X. Zhao Y. Wang L. Zhou L. (2013). Genome–wide survey by ChIP - seq reveals YY1 regulation of lincRNAs in skeletal myogenesis. *EMBO J.* 32 2575–2588. 10.1038/emboj.2013.182 23942234 PMC3791367

[B59] Man W. D. Kemp P. Moxham J. Polkey M. I. (2009). Skeletal muscle dysfunction in COPD: clinical and laboratory observations. *Clin. Sci. (Lond.)* 117 251–264. 10.1042/CS20080659 19681758

[B60] McCarthy J. J. Esser K. A. Peterson C. A. Dupont-Versteegden E. E. (2009). Evidence of MyomiR network regulation of beta-myosin heavy chain gene expression during skeletal muscle atrophy. *Physiol. Genomics* 39 219–226. 10.1152/physiolgenomics.00042.2009 19690046 PMC2789671

[B61] Mitra R. Adams C. M. Jiang W. Greenawalt E. Eischen C. M. (2020). Pan-cancer analysis reveals cooperativity of both strands of microRNA that regulate tumorigenesis and patient survival. *Nat. Commun.* 11:968. 10.1038/s41467-020-14713-2 32080184 PMC7033124

[B62] Narasimhan A. Ghosh S. Stretch C. Greiner R. Bathe O. F. Baracos V. (2017). Small RNAome profiling from human skeletal muscle: novel miRNAs and their targets associated with cancer cachexia. *J. Cachex. Sarcop. Muscle* 8 405–416. 10.1002/jcsm.12168 28058815 PMC5476855

[B63] Neppl R. L. Wu C. L. Walsh K. (2017). lncRNA Chronos is an aging-induced inhibitor of muscle hypertrophy. *J. Cell Biol.* 216 3497–3507. 10.1083/jcb.201612100 28855249 PMC5674882

[B64] O’Hern P. J. Do Carmo G. Gonçalves I. Brecht J. López Soto E. J. Simon J. (2017). Decreased microRNA levels lead to deleterious increases in neuronal M2 muscarinic receptors in Spinal Muscular Atrophy models. *Elife* 6:20752. 10.7554/eLife.20752 28463115 PMC5413352

[B65] Okugawa Y. Yao L. Toiyama Y. Yamamoto A. Shigemori T. Yin C. (2018). Prognostic impact of sarcopenia and its correlation with circulating miR-21 in colorectal cancer patients. *Oncol. Rep.* 39 1555–1564. 10.3892/or.2018.6270 29484416

[B66] Panguluri S. K. Bhatnagar S. Kumar A. McCarthy J. J. Srivastava A. K. Cooper N. G. (2010). Genomic profiling of messenger RNAs and microRNAs reveals potential mechanisms of TWEAK-induced skeletal muscle wasting in mice. *PLoS One* 5:e8760. 10.1371/journal.pone.0008760 20098732 PMC2808241

[B67] Paul R. Lee J. Donaldson A. V. Connolly M. Sharif M. Natanek S. A. (2018). miR-422a suppresses SMAD4 protein expression and promotes resistance to muscle loss. *J. Cachexia. Sarcopenia. Muscle* 9 119–128. 10.1002/jcsm.12236 28984049 PMC5803610

[B68] Ponnusamy M. Liu F. Zhang Y. Li R. Zhai M. Liu F. (2019). Long noncoding RNA CPR (cardiomyocyte proliferation regulator) regulates cardiomyocyte proliferation and cardiac repair. *Circulation* 139 2668–2684. 10.1161/CIRCULATIONAHA.118.035832 30832495

[B69] Porporato P. E. (2016). Understanding cachexia as a cancer metabolism syndrome. *Oncogenesis* 5:e200. 10.1038/oncsis.2016.3 26900952 PMC5154342

[B70] Prado C. M. Lieffers J. R. McCargar L. J. Reiman T. Sawyer M. B. Martin L. (2008). Prevalence and clinical implications of sarcopenic obesity in patients with solid tumours of the respiratory and gastrointestinal tracts: a population-based study. *Lancet Oncol.* 9 629–635. 10.1016/S1470-2045(08)70153-0 18539529

[B71] Qiu J. Wang L. Wang Y. Zhang Q. Ma W. Fang Q. (2018). MicroRNA351 targeting TRAF6 alleviates dexamethasone-induced myotube atrophy. *J. Thorac. Dis.* 10 6238–6246. 10.21037/jtd.2018.10.88 30622796 PMC6297431

[B72] Qiu J. Zhu J. Zhang R. Liang W. Ma W. Zhang Q. (2019). miR-125b-5p targeting TRAF6 relieves skeletal muscle atrophy induced by fasting or denervation. *Ann. Transl. Med.* 7:456. 10.21037/atm.2019.08.39 31700892 PMC6803201

[B73] Ramalho-Carvalho J. Martins J. B. Cekaite L. Sveen A. Torres-Ferreira J. Graca I. (2017). Epigenetic disruption of miR-130a promotes prostate cancer by targeting SEC23B and DEPDC1. *Cancer Lett.* 385 150–159. 10.1016/j.canlet.2016.10.028 27984115

[B74] Rom O. Reznick A. Z. (2016). The role of E3 ubiquitin-ligases MuRF-1 and MAFbx in loss of skeletal muscle mass. *Free Radical. Bio. Med.* 98 218–230. 10.1016/j.freeradbiomed.2015.12.031 26738803

[B75] Ross L. F. Kwon J. M. (2019). Spinal muscular atrophy: past, present, and future. *Neoreviews* 20 e437–e451. 10.1542/neo.20-8-e437 31371553

[B76] Rupaimoole R. Slack F. J. (2017). MicroRNA therapeutics: towards a new era for the management of cancer and other diseases. *Nat. Rev. Drug Discov.* 16 203–222. 10.1038/nrd.2016.246 28209991

[B77] Santer L. López B. Ravassa S. Baer C. Riedel I. Chatterjee S. (2019). Circulating long noncoding RNA LIPCAR predicts heart failure outcomes in patients without chronic kidney disease. *Hypertension* 73 820–828. 10.1161/HYPERTENSIONAHA.118.12261 30686085

[B78] Sartori R. Schirwis E. Blaauw B. Bortolanza S. Zhao J. Enzo E. (2013). BMP signaling controls muscle mass. *Nat. Genet.* 45 1309–1318. 10.1038/ng.2772 24076600

[B79] Shuai Y. Ma Z. Liu W. Yu T. Yan C. Jiang H. (2020). TEAD4 modulated LncRNA MNX1-AS1 contributes to gastric cancer progression partly through suppressing BTG2 and activating BCL2. *Mol. Cancer* 19:6. 10.1186/s12943-019-1104-1 31924214 PMC6953272

[B80] Silva W. J. Graça F. A. Cruz A. Silvestre J. G. Labeit S. Miyabara E. H. (2019). miR–29c improves skeletal muscle mass and function throughout myocyte proliferation and differentiation and by repressing atrophy - related genes. *Acta Physiol.* 226:13278. 10.1111/apha.13278 30943315 PMC6900115

[B81] Small E. M. O’Rourke J. R. Moresi V. Sutherland L. B. McAnally J. Gerard R. D. (2010). Regulation of PI3-kinase/Akt signaling by muscle-enriched microRNA-486. *Proc. Natl. Acad. Sci. U.S.A.* 107 4218–4223. 10.1073/pnas.1000300107 20142475 PMC2840099

[B82] Soares R. J. Cagnin S. Chemello F. Silvestrin M. Musaro A. De Pitta C. (2014). Involvement of MicroRNAs in the regulation of muscle wasting during catabolic conditions. *J. Biol. Chem.* 289 21909–21925. 10.1074/jbc.M114.561845 24891504 PMC4139209

[B83] Sonkoly E. Bata-Csorgo Z. Pivarcsi A. Polyanka H. Kenderessy-Szabo A. Molnar G. (2005). Identification and characterization of a novel, psoriasis susceptibility-related noncoding RNA gene, PRINS. *J. Biol. Chem.* 280 24159–24167. 10.1074/jbc.M501704200 15855153

[B84] Su Z. Xiong H. Pang J. Lin H. Lai L. Zhang H. (2019). LncRNA AW112010 promotes mitochondrial biogenesis and hair cell survival: implications for age-related hearing loss. *Oxid. Med. Cell Longev.* 2019 1–13. 10.1155/2019/6150148 31781342 PMC6855056

[B85] Sun L. Si M. Liu X. Choi J. M. Wang Y. Thomas S. S. (2018). Long-noncoding RNA Atrolnc-1 promotes muscle wasting in mice with chronic kidney disease. *J. Cachex. Sarcopenia Muscle* 9 962–974. 10.1002/jcsm.12321 30043444 PMC6204593

[B86] Sun M. Xia R. Jin F. Xu T. Liu Z. De W. (2014). Downregulated long noncoding RNA MEG3 is associated with poor prognosis and promotes cell proliferation in gastric cancer. *Tumor Biol.* 35 1065–1073. 10.1007/s13277-013-1142-z 24006224

[B87] Suzuki T. Springer J. (2018). MicroRNAs in muscle wasting. *J. Cachex. Sarcopenia Muscle* 9 1209–1212. 10.1002/jcsm.12384 30697980 PMC6351673

[B88] Tomlins S. A. Aubin S. M. Siddiqui J. Lonigro R. J. Sefton-Miller L. Miick S. (2011). Urine TMPRSS2:ERG fusion transcript stratifies prostate cancer risk in men with elevated serum PSA. *Sci. Transl. Med.* 3 72r–94r. 10.1126/scitranslmed.3001970 21813756 PMC3245713

[B89] Tomlins S. A. Day J. R. Lonigro R. J. Hovelson D. H. Siddiqui J. Kunju L. P. (2016). Urine TMPRSS2:ERG plus PCA3 for individualized prostate cancer risk assessment. *Eur. Urol.* 70 45–53. 10.1016/j.eururo.2015.04.039 25985884 PMC4644724

[B90] Valsecchi V. Anzilotti S. Serani A. Laudati G. Brancaccio P. Guida N. (2020). miR-206 reduces the severity of motor neuron degeneration in the facial nuclei of the brainstem in a mouse model of SMA. *Mol. Ther.* 28 1154–1166. 10.1016/j.ymthe.2020.01.013 32075715 PMC7132835

[B91] Wada S. Kato Y. Okutsu M. Miyaki S. Suzuki K. Yan Z. (2011). Translational suppression of atrophic regulators by microRNA-23a integrates resistance to skeletal muscle atrophy. *J. Biol. Chem.* 286 38456–38465. 10.1074/jbc.M111.271270 21926429 PMC3207415

[B92] Wang B. Zhang A. Wang H. Klein J. D. Tan L. Wang Z. (2019). miR-26a limits muscle wasting and cardiac fibrosis through exosome-mediated microRNA transfer in chronic kidney disease. *Theranostics* 9 1864–1877. 10.7150/thno.29579 31037144 PMC6485283

[B93] Wang B. Zhang C. Zhang A. Cai H. Price S. R. Wang X. H. (2017). MicroRNA-23a and MicroRNA-27a mimic exercise by ameliorating CKD-induced muscle atrophy. *J. Am. Soc. Nephrol.* 28 2631–2640. 10.1681/ASN.2016111213 28400445 PMC5576938

[B94] Wang H. Wang B. Zhang A. Hassounah F. Seow Y. Wood M. (2019). Exosome-mediated miR-29 transfer reduces muscle atrophy and kidney fibrosis in mice. *Mol. Ther.* 27 571–583. 10.1016/j.ymthe.2019.01.008 30711446 PMC6403486

[B95] Wang J. Cao B. Han D. Sun M. Feng J. (2017). Long Non-coding RNA H19 induces cerebral ischemia reperfusion injury via activation of autophagy. *Aging Dis.* 8:71. 10.14336/AD.2016.0530 28203482 PMC5287389

[B96] Wijesekara N. Konrad D. Eweida M. Jefferies C. Liadis N. Giacca A. (2005). Muscle-specific pten deletion protects against insulin resistance and diabetes. *Mol. Cell Biol.* 25 1135–1145. 10.1128/MCB.25.3.1135-1145.2005 15657439 PMC544010

[B97] Winbanks C. E. Beyer C. Hagg A. Qian H. Sepulveda P. V. Gregorevic P. (2013). miR-206 represses hypertrophy of myogenic cells but not muscle fibers via inhibition of HDAC4. *PLoS One* 8:e73589. 10.1371/journal.pone.0073589 24023888 PMC3759420

[B98] Woo C. J. Maier V. K. Davey R. Brennan J. Li G. Brothers J. (2017). Gene activation of SMN by selective disruption of lncRNA-mediated recruitment of PRC2 for the treatment of spinal muscular atrophy. *Proc. Natl. Acad. Sci. U.S.A.* 114 E1509–E1518. 10.1073/pnas.1616521114 28193854 PMC5338378

[B99] Worp W. R. P. H. Schols A. M. W. J. Dingemans A. M. C. Op Den, Kamp C. M. H. Degens J. H. R. J. (2020). Identification of microRNAs in skeletal muscle associated with lung cancer cachexia. *J. Cachex. Sarcopenia Muscle* 11 452–463. 10.1002/jcsm.12512 31828982 PMC7113505

[B100] Xu J. Li R. Workeneh B. Dong Y. Wang X. Hu Z. (2012). Transcription factor FoxO1, the dominant mediator of muscle wasting in chronic kidney disease, is inhibited by microRNA-486. *Kidney Int.* 82 401–411. 10.1038/ki.2012.84 22475820 PMC3393843

[B101] Yang X. Xue P. Chen H. Yuan M. Kang Y. Duscher D. (2020). Denervation drives skeletal muscle atrophy and induces mitochondrial dysfunction, mitophagy and apoptosis via miR-142a-5p/MFN1 axis. *Theranostics* 10 1415–1432. 10.7150/thno.40857 31938072 PMC6956801

[B102] Ydewalle D. Ramos D. M. Pyles N. J. Ng S. Gorz M. Pilato C. M. (2017). The antisense transcript SMN-AS1 regulates SMN expression and is a novel therapeutic target for spinal muscular atrophy. *Neuron* 93 66–79. 10.1016/j.neuron.2016.11.033 28017471 PMC5223741

[B103] Yin D. D. Liu Z. J. Zhang E. Kong R. Zhang Z. H. Guo R. H. (2015). Decreased expression of long noncoding RNA MEG3 affects cell proliferation and predicts a poor prognosis in patients with colorectal cancer. *Tumour Biol.* 36 4851–4859. 10.1007/s13277-015-3139-2 25636452

[B104] Yu Y. Li X. Liu L. Chai J. Haijun Z. Chu W. (2016). miR-628 promotes burn-induced skeletal muscle atrophy via targeting IRS1. *Int. J. Biol. Sci.* 12 1213–1224. 10.7150/ijbs.15496 27766036 PMC5069443

[B105] Zhang A. Li M. Wang B. Klein J. D. Price S. R. Wang X. H. (2018). miRNA-23a/27a attenuates muscle atrophy and renal fibrosis through muscle-kidney crosstalk. *J. Cachex. Sarcopenia Muscle* 9 755–770. 10.1002/jcsm.12296 29582582 PMC6104113

[B106] Zhang A. Wang H. Wang B. Yuan Y. Klein J. D. Wang X. H. (2019). Exogenous miR-26a suppresses muscle wasting and renal fibrosis in obstructive kidney disease. *FASEB J.* 33 13590–13601. 10.1096/fj.201900884R 31593640 PMC6894078

[B107] Zhang Z. K. Li J. Guan D. Liang C. Zhuo Z. Liu J. (2018a). Long noncoding RNA lncMUMA reverses established skeletal muscle atrophy following mechanical unloading. *Mol. Ther.* 26 2669–2680. 10.1016/j.ymthe.2018.09.014 30415659 PMC6225098

[B108] Zhang Z. K. Li J. Guan D. Liang C. Zhuo Z. Liu J. (2018b). A newly identified lncRNA MAR1 acts as a miR-487b sponge to promote skeletal muscle differentiation and regeneration. *J. Cachex. Sarcopenia Muscle* 9 613–626. 10.1002/jcsm.12281 29512357 PMC5989759

[B109] Zhou X. Yin C. Dang Y. Ye F. Zhang G. (2015). Identification of the long non-coding RNA H19 in plasma as a novel biomarker for diagnosis of gastric cancer. *Sci. Rep.UK* 5:11516. 10.1038/srep11516 26096073 PMC4476094

[B110] Zhu M. Liu J. Xiao J. Yang L. Cai M. Shen H. (2017). Lnc-mg is a long non-coding RNA that promotes myogenesis. *Nat. Commun.* 8:14718. 10.1038/ncomms14718 28281528 PMC5353601

[B111] Zhuang M. Gao W. Xu J. Wang P. Shu Y. (2014). The long non-coding RNA H19-derived miR-675 modulates human gastric cancer cell proliferation by targeting tumor suppressor RUNX1. *Biochem. Biophys. Res. Co.* 448 315–322. 10.1016/j.bbrc.2013.12.126 24388988

[B112] Zou T. Jaladanki S. K. Liu L. Xiao L. Chung H. K. Wang J. (2016). H19 long noncoding RNA regulates intestinal epithelial barrier function via MicroRNA 675 by interacting with RNA-binding protein HuR. *Mol. Cell Biol.* 36 1332–1341. 10.1128/MCB.01030-15 26884465 PMC4836219

